# Bone Loss in Diabetes Mellitus: Diaporosis

**DOI:** 10.3390/ijms25137269

**Published:** 2024-07-02

**Authors:** Krisztina Kupai, Hsu Lin Kang, Anikó Pósa, Ákos Csonka, Tamás Várkonyi, Zsuzsanna Valkusz

**Affiliations:** 1 Department of Internal Medicine, Albert Szent-Györgyi Medical School, University of Szeged, 6703 Szeged, Hungary; 2 Department of Oral Biology and Experimental Dental Research, Faculty of Dentistry, University of Szeged, 6703 Szeged, Hungary; 3 Department of Traumatology, University of Szeged, 6725 Szeged, Hungary; csonka.akos81@gmail.com

**Keywords:** diabetes mellitus, osteoporosis, antidiabetic medication

## Abstract

The objective of this review is to examine the connection between osteoporosis and diabetes, compare the underlying causes of osteoporosis in various forms of diabetes, and suggest optimal methods for diagnosing and assessing fracture risk in diabetic patients. This narrative review discusses the key factors contributing to the heightened risk of fractures in individuals with diabetes, as well as the shared elements impacting the treatment of both diabetes mellitus and osteoporosis. Understanding the close link between diabetes and a heightened risk of fractures is crucial in effectively managing both conditions. There are several review articles of meta-analysis regarding diaporosis. Nevertheless, no review articles showed collected and well-organized medications of antidiabetics and made for inconvenient reading for those who were interested in details of drug mechanisms. In this article, we presented collected and comprehensive charts of every antidiabetic medication which was linked to fracture risk and indicated plausible descriptions according to research articles.

## 1. Introduction

Two of the most significant illnesses afflicting people today are diabetes mellitus (DM) and osteoporosis, which put a strain on healthcare systems, drive up expenses, and shorten life expectancies [1,2]. 

A systemic skeletal disease called osteoporosis is defined as low bone mass and microarchitectural degeneration of bone tissue, which increases the fragility and fracture susceptibility of the bone. Changes in bone microarchitecture, bone matrix, and bone cell activity are influenced by certain aberrant turnover factors of bone mineral density (BMD). The impact of cortical or trabecular microarchitecture on the mechanical properties of bones cannot be fully concluded from pre-clinical research [3,4]. Because of their lower estrogen, postmenopausal women have been found to be more susceptible to this illness. Moreover, genes, long-term use of certain medications like corticosteroids, abnormal parathyroid hormone (PTH) levels, persistent alcohol and cigarette use, and a sedentary lifestyle are some of the many risk factors for the development of osteoporosis.

DM is a long-term metabolic disease brought on by insulin deregulation, which influences blood glucose levels. This condition is getting more dangerous due to inadequate management, increasing the chance of developing additional illnesses like heart disease, blindness, kidney failure, osteoporosis, and even death. Collected potential evidence suggests that both Type 1 and Type 2 DM (T1DM, T2DM) are associated with an increased risk of fractures, impacting bone growth and strength [3]. Both types exist non-enzymatic glycation of collagen by chronic hyperglycemia, which results in anomalies in the bone and an increased risk of fracture, particularly at the hip [5]. Elevated blood sugar can cause an osmotic reaction in osteoblasts, deteriorating the characteristics of bone material and leading to a higher risk of fragility fractures in individuals with T1DM and T2DM [6]. Meanwhile, the presence of both diabetes and osteoporosis can lead to greater health risks and mortality rates, so-called diaporosis or diabetoporosis, a secondary osteoporosis [7]. Additionally, individuals with diabetes are more likely to have vitamin D deficiencies, which further increases their risk of fractures [8]. 

It is difficult to predict fracture risk in persons with DM, since the disease has negative, fluctuating effects on bone. Identifying individuals who are at risk, addressing risk factors, selecting suitable medications, and utilizing clinically validated osteoporosis treatments are all part of prevention. Both intrinsic and extrinsic variables, such as low bone turnover, glycation end products, and microstructural alterations, can contribute to bone fragility. These elements raise the possibility of fragility fractures, especially in cases where aging populations pose a public health concern [9].

According to the World Health Organization (WHO), osteoporosis is characterized by reduced bone mass and BMD that is equal to or less than −2.5 standard deviations (SD) of the mean value for young, healthy individuals (a T-score ≤ −2.5 SD). A method of evaluating bone microarchitecture called dual energy X-ray absorptiometry (DXA) pictures is used to obtain the trabecular bone score. Quantitative computed tomography (QCT), high-resolution peripheral quantitative CT (HRpQCT), high-resolution magnetic resonance imaging (MRI), micro-CT, and hip structural analysis utilizing DXA are further techniques for evaluating bone health. Effective glucose management, preventing hypoglycemia and falls, engaging in exercise regimens to increase muscle and bone strength are general management strategies for osteoporosis in people with diabetes [6,10]. 

To date, there are no review articles which indicate comprehensive and systematic treatment of various anti-DM regarding diaporosis. In the present review article, we mentioned the fracture risk of DM and regular Fracture Risk Assessment Tool for monitoring BMD in DM patients briefly. Moreover, we showed the detail and plausible charts to emphasize on the molecular mechanisms of antidiabetic medications.

## 2. Basic Bone Cells

Osteocytes, bone lining cells, osteoblasts, and osteoclasts are types of bone cells. Mesenchymal stem cells give rise to osteoblasts, which initiate bone remodeling and create extracellular matrix on the surfaces of bones [11]. Through the modulation of osteoblast and osteoclast function, bone cells govern adaptive remodeling. 

**Figure 1:**
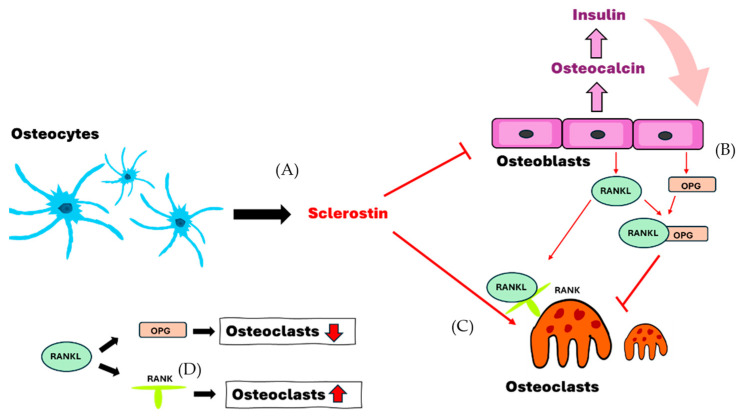
The interaction of osteocytes, osteoblasts and osteoclasts. (**A**) Osteocytes secrete sclerostin to induce the formation of osteoclasts and enhance the inhibition of osteoblasts. (**B**,**D**) Osteoblasts secrete OPG and RANKL and decrease the formation of osteoclasts in cases where both bind together. (**C**,**D**) Sclerostin and RANKL-RANK are able to enhance and assist the formation of osteoclasts. RANKL, receptor activator of nuclear factor-kappa B ligand; RANK, receptor activator of nuclear factor kappa B; OPG, osteoprotegerin.

Osteoblasts are cuboidal cells that form mineralized bone by producing and depositing extracellular matrix on bony surfaces. They react to tensile strains and express calcium channels. Collagen and other extracellular matrix proteins are secreted by osteoblasts. Large, multinucleated macrophage cells called osteoclasts help with bone resorption by attaching to the surface of the bone, forming an actin ring, and secreting acidic vesicles. A resorption pit is created when this process dissolves extracellular matrix and calcified bone [11]. Osteocytes, found in mineralized bone, are essential for detecting external mechanical loads and controlling adaptive remodeling [12]. They are found in a sophisticated system of tunnels known as canaliculi and within ovular chambers known as lacunae. Collagen and perlecan are examples of extracellular matrix components found in the lacunar canalicular network, which facilitates cell-to-cell communication. Osteocytes, which are terminally developed osteoblasts, can be identified by the expression of certain markers such as sclerostin (Figure 1), dentin matrix acidic phosphoprotein 1 (DMP1), fibroblast growth factor 23 (FGF23), and podoplanin [13,14,15,16]. The bone marrow is housed in the compact, strong cortical bone, which forms the outside edge of the skeleton [17]. It develops during embryonic osteogenesis, when osteoblasts during vascular invasion of the perichondrium construct the “bone collar”, which is the forerunner to the diaphyseal cortex. The material that makes up the bone collar is initially rather porous, but it eventually remodels to form a dense lamellar structure [18]. Osteoblasts regulate the cortical bone’s thickness and maturation, but osteoclasts are essential to the cortical formation process. 

## 3. Characteristics of Cortical Bone and Trabecular Bone in Brief

During embryogenesis and growth, the body’s structure is shaped by factors specific to the embryo [19,20]. Cortical bone comprises 80% of the adult skeleton, with the basic unit being the osteon or Haversian system [21,22]. Every osteon is positioned around a central Haversian canal that is lined with nerves, capillaries, venules, and arterioles. Osteocytes use canaliculi to interact with one another while they are imprisoned in lacunae. Compared to cancellous bone, cortical bone is stronger and denser and has a slower turnover rate [11,21,23]. Trabecular bone is a porous, heterogeneous, and anisotropic material found at the epiphyses of long bones and vertebral bodies [24,25]. Transferring loads from joints to the cortex of long bones, it is the primary load-bearing bone in vertebral bodies. Trabecular bone tissue is also composed of osteons, consists of both soft and hard tissue components, and is spongy and hierarchical [26]. Its mechanical properties are determined by its bone tissue composition and architecture. Compared to cortical bone, trabecular bone is composed of hydroxyapatite, collagen, and water, but it has lower calcium, tissue density, and ash fractions [27]. In comparison to cortical bone, it has a higher surface-to-volume ratio and significant bone remodeling. Its microstructural structure is made up of mineralized collagen fibrils with ellipsoid-shaped lacunae that are arranged in parallel lamellae and surrounded by cement lines [27,28,29,30].

## 4. Fracture Risk of T1DM

The hallmark of T1DM is nearly total insulin insufficiency, which lowers bone mass and raises the risk of fracture. In T1DM, insulin therapy stabilizes bone mass by increasing bone anabolic activity [5,31,32]. In osteoblasts, insulin activity promotes mitosis, suppresses apoptosis, and guards against the harmful effects of hyperglycemia on the development of new bone (Table 1). Amylin, which inhibits osteoclasts and increases osteoblasts, and insulin co-secretion are reduced when the pancreatic islets are destroyed by autoimmune disease [5,15,16]. BMD levels are lower in T1DM patients with nephropathy or neuropathy than in those without problems. Rather than more bone resorption, the primary skeletal change associated with T1DM is decreased bone production. Because insulin has an anabolic impact on bone, a low level of insulin production results in a low state of bone turnover [33]. Another peptide released by pancreatic beta-cells, amylin, is frequently reduced in T1DM and may have adverse effects on bone. A possible link between T1DM and a lower-quality bone structure is an increased risk of fractures. Low BMD, low bone formation markers such as osteocalcin, poor glycemic management, decreased physical activity, lower plasma insulin-like growth factor 1 (IGF-1), and celiac disease are all associated with T1DM in children and adolescents. Trabeculae in patients with concurrent microvascular disease and T1DM are thinner and more widely spaced. Aging bone material accumulates and the bone mineral matrix becomes more carbonated as a result of reduced bone turnover in T1DM [34].

Because osteoblast activity and differentiation are compromised, patients with T1DM have fragile bones. T1DM has an impact on osteoblast differentiation and function in the bone marrow, which lowers the quantity of mesenchymal stem cells and increases apoptosis. The inability to preserve pluripotent stem cells for osteoblast development is the mediating factor for this deficiency [33].

Osteocytes, which make up 90–95% of bone cells, are essential for bone remodeling and the development of fragile bone in diabetic individuals. They release sclerostin, a Wingless (Wnt) signaling pathway negative regulator that may affect diabetic patients’ bone quality [35]. According to a recent study, people with T2DM had higher sclerostin levels than those with T1DM, with a tendency for younger patients to have higher levels. The main cause of the bone fragility associated with T1DM is reduced bone formation, which may be caused by abnormalities in osteoclast activity or by communication between osteoblasts and osteoclasts [15]. Moreover, T1DM affects bone geometry and microarchitecture, affecting fracture risk and affecting bone structure [3]. Patients with diabetes experience reduced radial cortical, trabecular, and total surface area, which returns to normalized levels 5.5 years later [24,34,36,37].

## 5. Fracture Risk of T2DM

T2DM patients have normal or high BMD, but because of changes in the microarchitecture of the bone and a local humoral environment that promotes osteoclast activity, they are more likely to fracture [38] (Table 1). It is called the “diabetic paradox of bone fragility”. Changes in trabecular microstructure or cortical bone porosity are two indicators of bone strength that should be used to identify osteoporosis in people with T2DM. Rapid bone loss, decreased cortical density, variations in bone geometry, accumulation of microdamage in low bone turnover regions, and buildup of advanced glycation endproducts (AGEs) are among the factors that cause bone fragility in T2DM [39]. Increased inflammation and improved bone turnover may be linked to insulin resistance and an increase in adipose tissue [40,41]. Patients with T2DM frequently have low vitamin D levels, and aging-related pathogenetic pathways such elevated oxidative stress may play a role in the emergence of prevalent chronic diseases [42].

Reduced bone biomechanical qualities, decreased tissue yield strain, and decreased vertebral stiffness are the hallmarks of T2DM in postmenopausal women. In the presence of excessive glucose, T2DM is also linked to an increase in pro-inflammatory cytokines, which can result in decreased osteoblast viability and increased apoptosis. In comparison to non-diabetics and T2DM patients without fractures, this situation is also seen in patients who have previously fractured bones. Increased osteoblast apoptosis and decreased differentiation, decreased osteoclast differentiation, and altered osteocyte network and osteocytic mechanical responses are all consequences of T2DM on bone cells and matrix repair. Adipose tissue dysregulation and insulin resistance (IR) are two factors that lead to chronic low-grade inflammation, which can exacerbate bone loss. Patients with T2DM may have increased BMD for two main reasons: obesity and hyperinsulinemia. Increased fracture risk, limited bone turnover, and hyperglycemia in T2DM may all be associated with elevated sclerostin levels [43].

Compared to non-T2DM controls, postmenopausal women with T2DM exhibit higher cortical porosity. Despite increased BMD, the predicted risk of hip fractures is 2.1 for women and 2.8 for men. Patients with T2DM often experience sarcopenia, a loss of muscle mass and function, as a result of diabetes-related processes. Even if their BMDs range from normal to high, people with T2DM have a three times higher risk of hip fractures than people without the disease [19,20]. The paradoxical combination of increased bone fragility and retained BMD in these patients may be explained by changes in the microarchitecture of the bone, such as low cortical bone quality. One potential stand-in indicator of bone strength is the bone material strength index (BMSi). T2DM patients have complicated bone health because of conditions like obesity, hyperglycemia, retinopathy, and neuropathy. Obesity and hyperglycemia stimulate osteoclast-mediated resorption and interleukin-6 (IL-6), which in turn cause AGE deposition on collagen, decreased cross-linking of collagen and glycosuria, hypercalciuria, and a decrease in total body calcium. These factors are associated to bone abnormalities in T2DM. Patients with diabetes have higher serum levels of osteoprotegerin (OPG), which binds to receptor activator of nuclear factor-kappa B ligand (RANKL) [44]. Wnt/β-catenin pathway inactivation is another factor for reduced bone mass in diabetes [45,46,47].

## 6. Role of Advanced Glycation Endproducts (AGEs) in DM

Type 1 collagen and other bone proteins are susceptible to non-enzymatic glycosylation and the creation of AGEs, which can change the structure of the bone. Pentosidine is a fluorescent form of AGEs that can be measured using high-performance liquid chromatography. It builds up in connective tissues, including bone, as people age. By structurally changing collagen, AGEs can change the biomechanical characteristics of bone and reduce its elasticity. They inhibit osteoblast and osteoclast cell development in a dose-dependent way. Bone mechanical characteristics can deteriorate as a result of non-enzymatic glycation that produces AGEs inside the organic matrix. A spontaneous metabolic reaction between extracellular sugar and amino acid residues in the organic matrix results in the formation of AGEs.

Skeletal fragility in T2DM may be caused by decreased enzymatic cross-linking or an increase in non-enzymatic cross-links in the organic matrix, according to a diabetic mouse model. Decreased post-yield strain and toughness were linked to increased pentosidine, indicating that AGEs may exacerbate bone fragility in males with T2DM [48,49].

## 7. Bone Mineral Density (BMD)

Fractures in people with diabetes have a complicated and diverse pathophysiological process. The gold standard for diagnosing osteoporosis is BMD, although it only accounts for around 70% of bone strength. BMD is utilized in the diagnosis of osteoporosis, fracture risk assessment, and medication efficacy evaluation. The evaluation of fracture risk in diabetic individuals is still debatable, particularly in those with T2DM. Research continuously demonstrate that T1DM patients’ BMD is lower than that of non-diabetic controls. Due to its limited ability to capture changes in bone mass, BMD alone may understate the fracture risk in diabetic individuals. BMD is higher in T2DM patients than in age-matched non-diabetic people. The main components of bone strength are its structure and microarchitecture; bone mass, microarchitecture, and intrinsic material all play a role in the bone’s capacity to withstand fracture [3,50].

## 8. Diagnostic Tools for Osteoporosis

### 8.1. Dual-Energy X-ray Absorptiometry (DXA)

The gold standard for determining bone density and diagnosing osteoporosis is DXA, a two-dimensional projection technique. However, it frequently underestimates the risk of fracture in people with diabetes and is unable to properly capture changes in bone strength. The primary sites of measurement: the axial skeleton, lumbar spine, and proximal femur, which are vulnerable to lumbar degeneration and abdominal aortic calcification. All in all, DXA is the clinical gold standard, measures BMD in the general population, and accurately predicts fracture risk [51,52,53].

### 8.2. Quantitative Computed Tomography (QCT)

A noninvasive technique for assessing bone microstructure, including volume bone mineral density (vBMD), is QCT. It accurately converts QCT readings into the corresponding density of hydroxyapatite in a straightforward, practical, and noninvasive manner. Compared to DXA, QCT is more sensitive to changes in BMD caused by treatment or aging and can evaluate cortical and cancellous bones independently [54].

With minimal radiation exposure, HRpQCT may image and measure vBMD as well as bone microarchitecture, which includes cortical porosity. The distal tibia and distal radius are the typical sites for HRpQCT fracture prediction. Patients with T1DM had decreased cortical thickness and cortical vBMD at the ultra-distal tibia, according to a cross-sectional research [55].

It is hypothesized that patients with diabetes may have a higher fracture risk due to alterations in the distal tibia’s bone microstructure and a decline in vBMD. The non-weight-bearing distal radius may be associated with diabetic patients’ microcirculation disorders, such as neuropathy with length dependence. It is anticipated that HRpQCT will be an effective tool for evaluating fracture risk in diabetic patients; nevertheless, patients with various types and ages may present with distinct symptoms. T2DM is linked to cerebral impairments and retained trabecular characteristics, according to HRpQCT data [55,56].

### 8.3. The Fracture Risk Assessment Tool (FRAX)

The World Health Organization created the web-based FRAX to estimate the risk of osteoporotic fractures. It predicts the likelihood of hip and major osteoporotic fractures over the next ten years using clinical risk factors such as age, gender, height, body mass, prior fractures, parental hip fractures, smoking, glucocorticoids, rheumatoid arthritis, secondary osteoporosis, and excessive alcohol consumption. FRAX is not appropriate for those only with osteoporosis, fragility fractures, or anti-osteoporosis medications. Individuals with diabetes have a lesser risk prediction than those without diabetes, but those with diabetes have a higher fracture risk exactly. The FRAX score is similar to fracture risk despite these drawbacks [57,58,59].

### 8.4. Bone Histomorphometry

Bone histomorphometry is a technique that examines morphological and structural alterations in bone tissue sections using two-dimensional microscopic images. It may extract osteoid tissue area and volume as well as static and dynamic metrics of bone structure, including thickness, volume, and surface area. Additionally, it is capable of quantitatively analyzing the microstructure properties of bone, including the number of connecting points, trabecular bone area and thickness, bone production rate, and bone cortex thickness and porosity. In particular, bone histomorphometry is frequently utilized in the investigation and creation of medications for osteoporosis prevention and treatment. However, its use in patients with diabetes is limited due to the differing proportions of trabecular and cortical bone, which DXA scans cannot identify. Alternative imaging modalities are needed to identify these microarchitectural components, though their use is currently limited in clinical practice [60,61,62,63,64].

### 8.5. Microindentation

The primary types of microindentation that have been utilized to assess bone stiffness is reference point indention (RPI) and suggested to help in osteoporosis diagnosis. While bone density alone cannot predict fracture occurrence with sufficient accuracy, RPI measures mechanical characteristics directly and combines it with BMD assessment to produce a more accurate prognosis [65]. Understanding RPI parameters has been the subject of several reports [66]. Accumulated evidence has evaluated the connection between a patient’s BMSi and the likelihood of fracture using the OsteoProbe. These investigations, however, had different conclusions. One reported no significant correlation [67], while the other found that patients who had a fragility fracture had a considerably lower BMSi than patients who had no fracture [68].

## 9. Effects of Antidiabetic Treatments on Bone

### 9.1. Metformin

In human chorionic villous mesenchymal stem cells (CV-MSCs), metformin, an insulin sensitizer that lowers blood sugar, has been demonstrated to enhance osteogenesis through upregulating the expression of osteogenic genes such as runt-related transcription factor 2 (RUNX2), alkaline phosphatase (ALP), and osteopontin (OPN). Additionally, it activates the AMP-activated protein kinase (AMPK) signaling pathway in osteoblastic cells to promote differentiation and the formation of bone matrix [69]. Metformin inhibits bone resorption and the development of tartrate resistant acid phosphatase (TRAP)-positive multinucleated cells in bone marrow macrophage-derived osteoclasts in a dose-dependent manner. In a different investigation, metformin controlled the cytokine production in osteoblasts, which prevented osteoclast differentiation. As a first-line treatment for T2DM, metformin works by reducing the amount of glucose produced by the liver and inhibiting glucagon-mediated signaling in the liver. Preclinical research indicates that metformin has a direct osteogenic effect via activating AMPK, which influences the growth and differentiation of osteoblasts and osteoclasts as well as the production of other biochemical factors [70]. Preclinical research has shown that metformin enhances insulin sensitivity and positively impacts bone mineral density. Gene expression, including that of peroxisome proliferator-activated receptor gamma (PPARγ), is decreased by AMPK activation, which prevents adipogenesis. Additionally, it decreases bone resorption by inhibiting nuclear factor of activated T cells 1(NFATc1) and increases RUNX2 to promote bone growth. Moreover, AMPK activation suppresses osteoclastogenesis and reduces the expression of receptor activator of nuclear factor-kappa-B ligand (RANKL) [71]. The way that metformin affects the skeletal system is by secreting OPG and preventing the expression of RANKL, which changes the OPG/RANKL axis [72]. Bone microarchitecture, bone mineral density, and bone remodeling are all determined by this change in the OPG/RANKL ratio [73].

Clinical studies on metformin show that it increases BMD and decreases bone turnover, with a neutral effect on fracture risk in T2DM patients. Metformin was associated with a 19% reduced fracture risk in T2DM patients [74,75,76]. 

**Figure 2:**
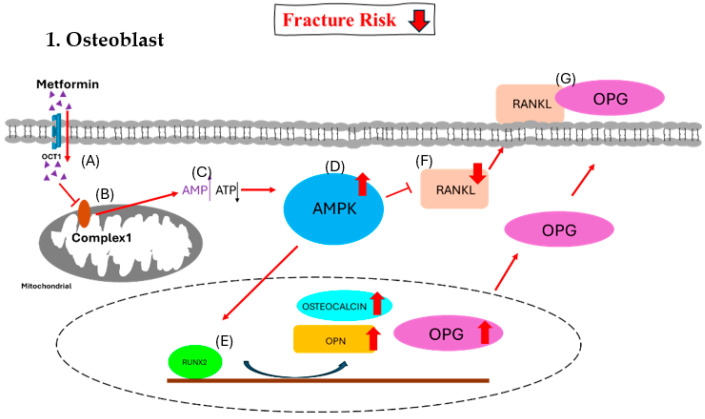
Effect of metformin on osteoblast. (**A**) Metformin enters the osteoblast via OCT1. (**B**) Metformin inhibits the function of mitochondrial respiratory complex 1. (**C**) The inhibition causes increased [AMP]-to-[ATP] ratio signals. (**D**) The higher ratio triggers the activation of the AMPK complex. (**E**) The AMPK complex activates RUNX2 and triggers an increase in osteocalcin, OPN, and OPG. (**F**) The AMPK complex decreases RANKL, and (**G**) most of OPG binds with RANKL together to inhibit the formation of osteoclasts.

**Figure 3:**
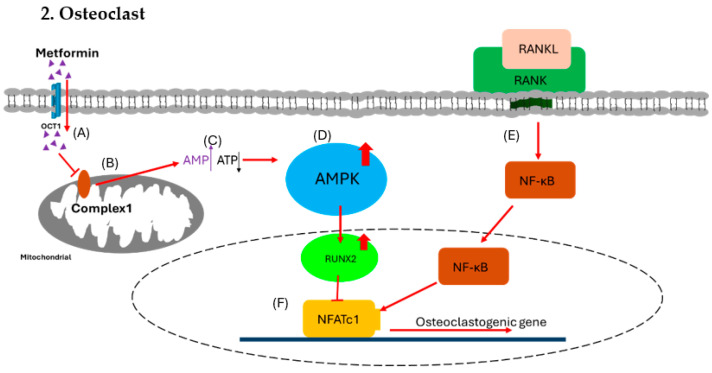
Effect of metformin on osteoclast. (**A**) Metformin enters osteoblast via OCT1. (**B**) The metformin inhibits the function of mitochondrial respiratory complex 1. (**C**) The inhibition causes increased [AMP] to [ATP] ratio signals. (**D**) The higher ratio triggers the activation of the AMPK complex. (**E**) In the meantime, RANKL binds with RANK to trigger NF-κB for osteoclastogenesis. (**F**) The AMPK complex activates RUNX2, which inhibits NFATc1 to block osteoclastogenesis and reduce the formation of osteoclast.

For diabetics with weak bones, metformin is the recommended medication; nevertheless, it is unknown how directly it affects osteoblast development and proliferation. It is possible that applying metformin to bone cell cultures in high-glucose or AGE-containing environments has no bearing on the medication’s real therapeutic benefits in diabetes patients. The most severely impacted cells in diabetic bone disease, osteocytes, are not yet understood to be altered by metformin. Concentrated study is required to ascertain the true effects of metformin on bone as statins, which have initially demonstrated anabolic effects on bone, may only partially reduce the incidence of fracture [77,78,79,80] (Figure 2 and Figure 3).

OCT1, organic cation transporter 1; AMPK, AMP-activated protein kinase; RUNX2, runt-related transcription factor 2; RANKL, receptor activator of nuclear factor-kappa B ligand; RANK, receptor activator of nuclear factor kappa B; OPN, osteopontin; OPG, osteoprotegerin.

OCT1, organic cation transporter 1; AMPK, AMP-activated protein kinase; RUNX2, runt-related transcription factor 2; RANKL, receptor activator of nuclear factor-kappa B ligand; RANK, receptor activator of nuclear factor kappa B; NFATc1, nuclear factor of activated T cells 1.

### 9.2. Sulfonylureas

Sulfonylureas (SUs) are non-insulin glucose-lowering drugs used in managing T2DM [81]. They are classified into first, second, and third generation agents. SUs are used by 50–80% of diabetic patients worldwide [82]. Large clinical trials have validated the effectiveness and acceptable safety of new-generation SUs in the control of diabetes, despite possible hypoglycemia consequences [83].

By inducing pancreatic beta cells to secrete more insulin, SUs are used to treat T2DM. They reduce blood glucose levels and somewhat alleviate insulin insufficiency by inducing glucose-dependent insulin production [84]. When the K_ATP_ channel is blocked, intracellular K^+^ ions accumulate, depolarizing the cell’s inner membrane and attracting extracellular calcium ions [85]. These calcium ions bind to insulin vesicles, promoting insulin release into the circulation. Sulfonylureas also play a role in glucose control by combining and closing the K_ATP_ channel, leading to depolarization and opening of voltage-gated calcium channels [86,87].

**Figure 4:**
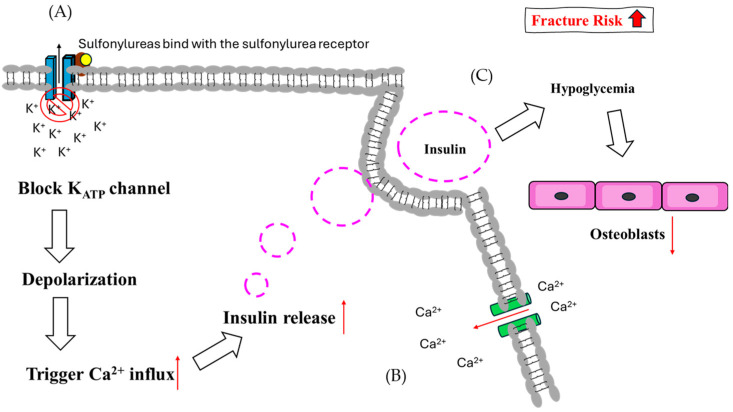
The theoretical mechanism of sulfonylureas regarding fracture risk. (**A**) In pancreatic cells, SUs bind with SU receptors and block K_ATP_ channels, causing depolarization. (**B**) Following depolarization, Ca^2+^ influx is induced and enhances the release of insulin. (**C**) Hypoglycemia suppresses osteoblast function due to glucose supply sensitivity.

SUs trigger insulin secretion and then cause hypoglycemia, which may increase the risk of fractures in patients with T2DM [88]. The function of SUs in bone metabolism is still unclear, but recent evidence suggests that the risk of hip fracture in treated patients is almost double due to higher hypoglycemic rates. In T2DM patients, SU treatment has been linked to a 14% increase in fracture risk, which was lower than insulin, higher than metformin, and comparable to thiazolidinedione [89]. SU use is significantly associated with fracture risk, and initial therapy should be undertaken prudently in both men and women [85,90] (Figure 4).

### 9.3. Thiazolidinedione

2,4-Thiazolidinediones (TZDs) are selective PPARγ agonists that modulate glucose and lipid metabolism genes [91]. These drugs are the first to address insulin resistance in T2DM patients [92]. TZDs have various effects, including antimicrobial, antiviral, antioxidant, anticancer, anti-inflammatory, anti-plasmodial, and anti-hyperglycemic effects [93]. They are also known to lower fasting and postprandial glucose concentrations and free fatty acid concentrations, indicating that they act as insulin sensitizers [94]. However, their use has been limited due to their potential adverse events, such as fluid retention, heart failure, and increased fracture risk [95,96]. Peroxisome proliferator-activated receptors (PPARs) are nuclear receptors in the body that regulate transcription of genes involved in gluconeogenesis, lipid transport, and fatty acid oxidation [97]. Activation of PPARγ in adipocytes decreases inflammatory cytokines and free fatty acids, improving insulin sensitivity. PPARγ is essential for adipocyte differentiation, proliferation, and fatty acid uptake and storage [98]. TZDs activate PPARγ, forming a heterodimer with the retinoid X receptor (RXR) and recognizing specific DNA response elements. PPARγ agonists can reduce insulin resistance, decrease hepatic gluconeogenesis, and reduce blood glucose levels. Although TZDs have not been widely accepted for T2DM therapy, their pleiotropic actions make them appealing [99].

**Figure 5:**
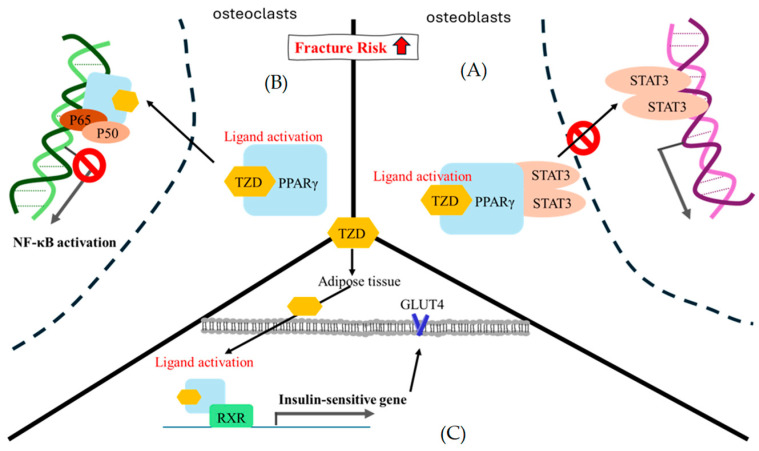
The theoretical mechanism of thiazolidinedione regarding fracture risk. (**A**) In osteoblasts, STAT3 is likely to mediate osteoblast differentiation. The ligand activation of TZD-PPARγ binding encourages STAT3 recruitment and pauses STAT3-DNA binding. (**B**) In osteoclasts, NF-κB is an important factor in osteoclast formation, and p50/p60 is a key point heterodimer for NF-κB signal activation. At present, there are several assumptions regarding the gene trans-repression mechanism of PPARγ. Here, we only showed the direct physical interaction in which ligand activation of TZD-PPARγ binding interacted with the p50/p60 heterodimer to pause NF-κB signal activation [97]. (**C**) In adipose cells, the ligand activation of TZD-PPARγ binding is prone to interact with RXR and enhance a series of insulin-sensitive genes, such as GLUT4. RXR, retinoid X receptor; GLUT4, glucose transporter type 4; STAT3, signal transducer and activator of transcription 3.

TZDs are insulin sensitizers that have been linked to increased bone marrow adipocytes, a risk factor for fractures and bone loss. Because TZDs decrease osteoclast-specific transcription factor activity and osteoblast-specific signaling pathway activity, they decrease bone mineral density and raise the risk of fracture [100]. They also inhibit osteogenesis, increasing the risk of osteoporosis in diabetes patients. TZDs have also been found to negatively impact bone formation and resorption [101]. The use of TZDs is particularly harmful in postmenopausal rats, a risk factor for diabetes and osteoporosis [102]. Changes in PPARγ, however, can preserve the beneficial effects on energy metabolism while lessening the detrimental effects on bone metabolism [103] (Figure 5).

### 9.4. Incretin System Modulation: GLP-1 Receptor Agonists (GLP-1 RAs)

Incretin hormones, released after meal ingestion, accelerate glucose metabolism by triggering insulin secretion from the pancreas. These hormones influence the synthesis and resorption of bone as well as the expression of bone markers. These are elements that stimulate the release of insulin when glucose is consumed. The two main incretins secreted from the gut are glucagon-like peptide-1(GLP-1) and glucose-dependent insulinotropic polypeptide (GIP), which are activated by dietary intake and function through incretin receptors [104]. Serum levels of the bone resorption marker do not drop following GIP and GLP-1 administration. 

**Figure 6:**
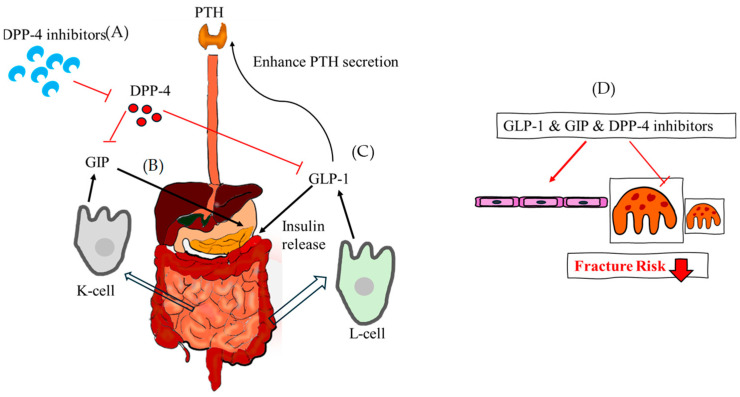
The theoretical mechanism of GLP-1, GIP and DPP-4 inhibitor regarding fracture risk. (**A**) DPP-4 inhibitors reduce the function of DPP-4 and increase GIP and GLP-1 indirectly. (**B**) GIP is released from the K cells of the small intestine and stimulates the release of insulin. (**C**) GLP-1, which is released from the L-cell of the intestine at low basal levels, also stimulates the release of insulin and enhances PTH secretion. (**D**) Taken together, GLP-1, GIP, and DPP-4 inhibitors assist in the formation of osteoblasts, reducing the fracture risk. DPP-4, dipeptidyl pepetidase-4; GLP-1, glucagon-like peptide-1; GIP, glucose-dependent insulinotropic polypeptide.

Initially identified as gastric inhibitory polypeptide, the first incretin hormone was subsequently renamed GIP when it was extracted from crude extracts of the small intestine of pigs. When fat and glucose are consumed, the small intestine’s K-cells release GIP, a 42 amino acid peptide hormone. Via a particular GIP receptor, it increases the amount of insulin secreted in response to glucose. Pancreatic cells have been found to contain GIP, indicating an intra-islet cell-to-cell communication [105]. In vitro, osteoblast apoptosis is decreased by GIP stimulation, which also increases intracellular cAMP levels, cell survival, and type 1 collagen expression. When nutrients are ingested, intestinal L-cells release this tissue-specific posttranslational proteolytic product, which increases human glucose-stimulated insulin production. In the fasting and interprandial states, GLP-1 is continuously released from the intestine at low basal levels; following food absorption, circulating levels increase two- to three-fold. It has several functions in maintaining metabolic homeostasis, including promoting insulin production and glucose-dependent insulin secretion, preventing gastric emptying and glucagon release, and reducing appetite. T2DM can be effectively treated with GLP-1 receptor agonists (GLP-1RAs). Enteroendocrine cells of the intestinal mucosa release GLP-1; however, GLP-1 receptor is extensively expressed by many cell types, including islet beta-cells, and they affect metabolism in different organs. Exendin-4, a GLP-1RA, has been demonstrated to reduce osteoclast production and bone resorption in vivo in a mouse model of inflammation generated by lipopolysaccharide. Additionally, bone-related cells such as osteoblasts, osteocytes, and osteoclasts express GLP-1 receptors [106]. Bone marrow stem cells and adipose-derived stem cells also express GLP-1 receptors and upregulated during osteoblast differentiation [107]. In diabetic rats, GLP-1 administration has been demonstrated to have positive effects on trabecular separation and trabecular bone pattern factor (TBPf) [108]. In diabetic animal models, treatment with GLP-1RA has been shown to stop bone loss. Peptide analogue of GLP-1, exenatide, having a prolonged plasma half-life because dipeptidyl pepetidase-4 (DPP-4) cannot break it down. In an animal model of periodontitis, treatment with liraglutide improves ligature-induced alveolar bone resorption and decreases osteoclasts on the alveolar bone surface [109] (Figure 6).

### 9.5. Incretin System Modulation: Dipeptidyl Pepetidase-4 (DPP-4) Inhibitors

DPP-4 inhibitors are used to treat T2DM by inhibiting the degradation of incretins. These oral anti-hyperglycemic agents augment the biological activity of incretin hormones, restoring many diabetic pathophysiological problems [110]. Furthermore, DPP-4 has been connected to a number of pathogenic processes, such as viral entry, inflammation, immune-mediated illnesses, and tumor biology. It is a 110 kDa transmembrane-spanning glycoprotein exopeptidase that is highly accessible to peptide substrates and is expressed in a variety of tissues, including endothelial cells [111]. 

When taken orally, once or twice a day, DPP-4 inhibitors significantly reduce plasma DPP-4 activity in about five minutes. The kidney is the main organ responsible for their elimination with high renal clearance of glomerular filtration. DPP-4 inhibitors improve glucose-stimulated insulin production and physiologic glucose regulation by preventing the breakdown of GLP-1 and GIP [112]. They have a favorable tolerability profile and pharmacokinetic and pharmacodynamic qualities and may be helpful in treating T2DM [113]. DPP-4 inhibitors may lower hypoglycemia risk but have conflicting long-term benefits. In general, these may be good initial therapies for patients at risk for hypoglycemia [112]. The prolyl oligo-peptidase/serine peptidase gene family includes DPP-4. DPP-4 inhibitors can decrease PTH levels, inhibit calcium release, and increase serum vitamin D3 concentration, promoting bone growth and remodeling. Sitagliptin, a type of DPP-4 inhibitor, affects bone turnover markers. It also promotes insulin secretion, improving glucose tolerance, and reducing the negative effects of hyperglycemia on bone. The higher plasma DPP-4 activity in obese people could be explained by the release of DPP-4 from adipose tissue. Visceral adipocytes express human CD26/DPP-4, a type 2 transmembrane serine protease with 766 amino acids, greater than other cell types [114]. DPP-4 inhibitors are also increasingly used to manage T2DM patients at an increased risk of fractures [115]. Patients using DPP-4 inhibitors had a lower fracture risk than those on other diabetic drugs, according to a meta-analysis of clinical trials. Inhibiting DPP-4 in MKR mice did not change turnover, bone microarchitecture, or glycemia, according to one study [116]. The possible advantages of DPP-4 inhibitors for bone are mediated through indirect processes rather than direct interactions with osteoblasts [117]. All in all, DPP-4 inhibitors can protect bone and reduce fracture risk particularly sitagliptin. They improve bone mineral density, quality, and markers [118] (Figure 6).

### 9.6. Sodium–Glucose Cotransporter 2 Inhibitors (SGLT2i)

SGLT-2i are glucose-lowering agents that inhibit glucose reuptake at the renal proximal tubule, leading to glycosuria and reduced plasma glucose [119]. By decreasing renal tubular glucose reabsorption, SGLT-2i lowers blood sugar levels without inducing the release of insulin. SGLT-2i can be used in patients with long-standing diabetes and mostly expressed in the proximal renal tubules and show dose-dependent glucosuria and blood glucose reduction in T2DM [120].

**Figure 7:**
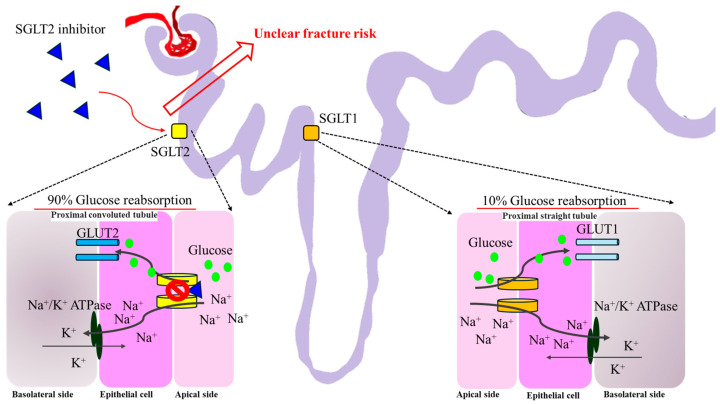
The theoretical mechanism of SGLT-2i regarding fracture risk. Most of the reabsorbed glucose in the renal proximal tubule is via SGLT2. Approximately 10% of glucose reabsorption via SGLT1 occurs at the proximal straight tubule. SGLT-2i occupies SGLT2 and blocks the entry of glucose through GLUT2 indirectly. To date, there is still an uncertain fracture risk for SGLT-2i based on clinical data. SGLT1, sodium–glucose cotransporter 1; SGLT2, sodium–glucose cotransporter 2; GLUT1, glucose transporter type 1; GLUT2, glucose transporter type 2.

SGLT-2i may alter calcium and phosphate homeostasis, potentially and theoretically increasing the risk of bone fracture. Older patients with preexisting microvascular diseases, impaired baseline renal function, and higher baseline risk of fall are at higher risk [121]. SGLT-2i also increase serum phosphate, leading to increased fibroblast growth factor-23 and PTH, causing osteomalacia [122]. However, dapagliflozin and empagliflozin do not seem to have an impact on the frequency of fractures in clinical trials [123]. The exact pathogenetic mechanism by which SGLT-2i raise the risk of fractures is unknown [124]. There is no correlation between the frequency of different site-specific fractures and the usage of SGLT-2i [125]. Use of SGLT-2i is not associated with an increased risk of nonvertebral fractures or fractures in general, and no association has been shown between the use of SGLT-2i is and the incidence of different site-specific fractures in these cohort studies [126] (Figure 7).

### 9.7. Insulin

Insulin is a hormone that regulates blood glucose levels by affecting macronutrient metabolism and cellular glucose transport [48]. It is produced by pancreatic beta cells in response to glucose, while IGF-1 is synthesized by the liver [127]. Insulin and IGF-1 exhibit a decreased affinity for binding and activating each other’s receptors. Osteoblasts express functional insulin receptor and respond to exogenous insulin by increasing bone anabolic markers. IGF-1 can bind insulin receptor and activate insulin receptor substrate (IRS), which is a downstream substrate in osteoblasts [44]. IRS is crucial for insulin and IGF-1 signal transduction, and insulin signaling in osteoblasts promotes osteocalcin carboxylation [50].

Insulin signaling in osteoblasts regulates osteocalcin production and bioavailability. The insulin receptor is expressed by osteoblasts and osteoclast-like cells, and the reduction in bone turnover after insulin infusion is likely related to hypoglycemia, which suppresses osteoclast and osteoblast function due to glucose supply sensitivity and hyperinsulinemia reduces PTH secretion, affecting insulin-induced hypoglycemia [128,129,130]. 

Additionally, abdominal obesity and T2DM are linked to insulin resistance, low bone turnover, and increased fracture risk [49]. Serum calcium levels and spine bone mineral density are frequently greater in those with T2DM and abdominal obesity. All in all, insulin and IGF-1 influence bone mass variability and promote bone formation by circulating to osteoblasts [131] (Figure 1).

## 10. Discussion

Diabetes mellitus is linked to a higher risk of fragility fractures. Patients with diabetes are evaluated for fracture risk using a variety of techniques, including BMD, FRAX, sclerostin, HR-pQCT, microindentation, and BTMs. However, because of complex pathophysiological pathways, no single technique is ideal for all circumstances, especially in T2DM.

Insulin is essential for the anabolic impact of insulin on osteoblasts. Hyperglycemia is a common symptom of both T1DM and T2DM, with T1DM primarily having insulin deficiency and T2DM patients having insulin resistance. Low levels and/or action of IGF-1 are commonly linked to insulin-deficient circumstances in T1DM, which can lead to low peak bone mass at an early age.

The most notable aspect of T2DM is the emergence of insulin resistance, which helps to explain why BMD is either normal or higher in these groups. On the other hand, it can also worsen the quality of the bone by causing osteoblasts to become resistant to the effects of IGF-1 and AGE levels to rise. These factors might raise the risk of fractures, oxidative stress, and damage to the bone matrix.

AGEs have a negative correlation with BMSi, particularly in T2DM. They also have the ability to attach to the transmembrane protein receptor for AGE (RAGE), which is partially located in the osteoclastic and osteoblastic cell lineages and uses signal transduction to control bone production and resorption. 

T2DM is a disorder that results in decreased bone strength due to compromised microarchitectural and structural alterations, but minimal bone turnover and retained BMD. Because existing fracture risk predictors rely on BMD, they understate the influence of T2DM on fracture risk. When comprehensive diabetes-related data and fractures are gathered concurrently in cohorts, better fracture prediction research is required. Both bone and glycemic status should be taken into account when selecting drugs. Although T1DM and T2DM are distinct conditions, both kinds of diabetes may be caused by similar biological processes. Maintaining adequate glucose control is essential for shielding patients from the effects of diabetes [132].

Anti-diabetic medications may affect bone metabolism negatively, favorably, or neutrally. Notably, clinical data frequently contradict the findings of experimental investigations [48]. This article is also intended to look into the relationship between DM and antidiabetic medications (metformin, SUs, TZDs, GLP-1 RAs, DPP-4 inhibitors, SGLT-2i, and insulin) and their respective risks of fracture [122]. According to significant evidence, we concluded that DM patients are not likely to have a higher risk of fracture if they take metformin, GLP-1 RAs, or DPP-4 inhibitors.

## 11. Conclusions

In conclusion, diaporosis fractures are a serious risk factor for patients with DM. When assessing fracture risk in patients with DM, traditional BMD tests like DXA and FRAX often underestimate the risk, but TBS can indirectly quantify changes in bone microstructure. Diabetes-related skeletal fragility is varied and has a substantial clinical impact. A bone-centric approach identifies important gaps in the diagnosis and treatment of individuals with diabetic bone disease. Poorer skeletal results are linked to and may be exacerbated by metabolic abnormalities. It may be possible to resolve current discrepancies by incorporating diabetes-specific factors for skeletal assessment, especially with reference to the pathophysiological mechanisms regarding diaporosis. 

## Figures and Tables

**Table 1:** The characteristics of fracture risks within T1DM and T2DM.

Bone Icon	T1DM	T2DM
Bone strength	Cortical stability ↓ Trabecular stability ↓ Bending resistance↓ Stiffness ↓	Cortical stability ↑ Trabecular stability ↓ Bending resistance ↓ Stiffness ↓
Microarchitecture	Cortical porosity ↑ Cross-sectional area ↓ TBS ↓	Cortical porosity ↑ Cross-sectional area ↓ TBS ↓
Bone turnover	Bone turnover ↓ Sclerostin level ↑ Bone turnover marker ↓	Bone turnover ↓ Sclerostin level ↑ Bone turnover marker ↓
BMD	BMD ↓ Cortical volumetric BMD ↓	BMD normal or ↑ Cortical volumetric BMD ↓

TBS, trabecular bone score; BMD, bone mineral density.

## Abbreviation

OCT1	organic cation transporter 1
AMPK	AMP-activated protein kinase
RUNX2	runt-related transcription factor 2
RANKL	receptor activator of nuclear factor-kappa B ligand
RANK	receptor activator of nuclear factor kappa B
OPN	osteopontin
OPG	osteoprotegerin.
NFATc1	nuclear factor of activated T cells 1.

## References

[1] Heilmeier U., Patsch J. (2016). Diabetes and Bone. Semin. Musculoskelet. Radiol..

[2] Araújo I.M.D., Moreira M.L.M., Paula F.J.A.D. (2022). Diabetes and Bone. Arch. Endocrinol. Metab..

[3] Palui R., Pramanik S., Mondal S., Ray S. (2021). Critical Review of Bone Health, Fracture Risk and Management of Bone Fragility in Diabetes Mellitus. World J. Diabetes.

[4] Guo C.-J., Xie J.-J., Hong R.-H., Pan H.-S., Zhang F.-G., Liang Y.-M. (2019). Puerarin Alleviates Streptozotocin (STZ)-Induced Osteoporosis in Rats through Suppressing Inflammation and Apoptosis via HDAC1/HDAC3 Signaling. Biomed. Pharmacother..

[5] Merlotti D., Gennari L., Dotta F., Lauro D., Nuti R. (2010). Mechanisms of Impaired Bone Strength in Type 1 and 2 Diabetes. Nutr. Metab. Cardiovasc. Dis..

[6] Chen W., Mao M., Fang J., Xie Y., Rui Y. (2022). Fracture Risk Assessment in Diabetes Mellitus. Front. Endocrinol..

[7] Ebeling P.R., Nguyen H.H., Aleksova J., Vincent A.J., Wong P., Milat F. (2022). Secondary Osteoporosis. Endocr. Rev..

[8] Schacter G.I., Leslie W.D. (2021). Diabetes and Osteoporosis. Endocrinol. Metab. Clin. N. Am..

[9] Hofbauer L.C., Busse B., Eastell R., Ferrari S., Frost M., Müller R., Burden A.M., Rivadeneira F., Napoli N., Rauner M. (2022). Bone Fragility in Diabetes: Novel Concepts and Clinical Implications. Lancet Diabetes Endocrinol..

[10] Chandra A., Rajawat J. (2021). Skeletal Aging and Osteoporosis: Mechanisms and Therapeutics. Int. J. Mol. Sci..

[11] Jolly J.J., Chin K.-Y., Farhana M.F.N., Alias E., Chua K.H., Hasan W.N.W., Ima-Nirwana S. (2018). Optimization of the Static Human Osteoblast/Osteoclast Co-Culture System. Iran. J. Med. Sci..

[12] Aglan H.A., Kotob S.E., Mahmoud N.S., Kishta M.S., Ahmed H.H. (2024). Bone Marrow Stem Cell-Derived β-Cells: New Issue for Diabetes Cell Therapy. Tissue Cell.

[13] Martiniakova M., Biro R., Kovacova V., Babikova M., Zemanova N., Mondockova V., Omelka R. (2024). Current Knowledge of Bone-Derived Factor Osteocalcin: Its Role in the Management and Treatment of Diabetes Mellitus, Osteoporosis, Osteopetrosis and Inflammatory Joint Diseases. J. Mol. Med..

[14] Schröder K. (2015). NADPH Oxidases in Bone Homeostasis and Osteoporosis. Cell. Mol. Life Sci..

[15] Catalano A., Pintaudi B., Morabito N., Di Vieste G., Giunta L., Bruno M.L., Cucinotta D., Lasco A., Di Benedetto A. (2014). Gender Differences in Sclerostin and Clinical Characteristics in Type 1 Diabetes Mellitus. Eur. J. Endocrinol..

[16] Neumann T., Hofbauer L.C., Rauner M., Lodes S., Kästner B., Franke S., Kiehntopf M., Lehmann T., Müller U.A., Wolf G. (2014). Clinical and Endocrine Correlates of Circulating Sclerostin Levels in Patients with Type 1 Diabetes Mellitus. Clin. Endocrinol..

[17] Black J.D., Tadros B.J. (2020). Bone Structure: From Cortical to Calcium. Orthop. Trauma.

[18] Isojima T., Sims N.A. (2021). Cortical Bone Development, Maintenance and Porosity: Genetic Alterations in Humans and Mice Influencing Chondrocytes, Osteoclasts, Osteoblasts and Osteocytes. Cell. Mol. Life Sci..

[19] Samakkarnthai P., Sfeir J.G., Atkinson E.J., Achenbach S.J., Wennberg P.W., Dyck P.J., Tweed A.J., Volkman T.L., Amin S., Farr J.N. (2020). Determinants of Bone Material Strength and Cortical Porosity in Patients with Type 2 Diabetes Mellitus. J. Clin. Endocrinol. Metab..

[20] Wölfel E.M., Fiedler I.A.K., Dragoun Kolibova S., Krug J., Lin M.-C., Yazigi B., Siebels A.K., Mushumba H., Wulff B., Ondruschka B. (2022). Human Tibial Cortical Bone with High Porosity in Type 2 Diabetes Mellitus Is Accompanied by Distinctive Bone Material Properties. Bone.

[21] Heinemann C., Heinemann S., Worch H., Hanke T., Max Bergmann Center of Biomaterials and Institute of Materials Science, Dresden University of Technology (2011). Development of an Osteoblast/Osteoclast Co-Culture Derived by Human Bone Marrow Stromal Cells and Human Monocytes for Biomaterials Testing. Eur. Cell. Mater..

[22] Harper K., Sathiadoss P., Saifuddin A., Sheikh A. (2021). A Review of Imaging of Surface Sarcomas of Bone. Skeletal Radiol..

[23] Li Z., Yue M., Zhou Y. (2024). Advances in Material-Based Strategies for Diabetic Bone Regeneration. Stem Cells Transl. Med..

[24] Abdalrahaman N., McComb C., Foster J.E., McLean J., Lindsay R.S., McClure J., McMillan M., Drummond R., Gordon D., McKay G.A. (2015). Deficits in Trabecular Bone Microarchitecture in Young Women With Type 1 Diabetes Mellitus. J. Bone Miner. Res..

[25] Callens S.J.P., Tourolle Né Betts D.C., Müller R., Zadpoor A.A. (2021). The Local and Global Geometry of Trabecular Bone. Acta Biomater..

[26] Kong S.H., Hong N., Kim J.-W., Kim D.Y., Kim J.H. (2021). Application of the Trabecular Bone Score in Clinical Practice. J. Bone Metab..

[27] O’Connor D.T., Elkhodary K.I., Fouad Y., Greene M.S., Sabet F.A., Qian J., Zhang Y., Liu W.K., Jasiuk I. (2016). Modeling Orthotropic Elasticity, Localized Plasticity and Fracture in Trabecular Bone. Comput. Mech..

[28] Sieberath A., Della Bella E., Ferreira A.M., Gentile P., Eglin D., Dalgarno K. (2020). A Comparison of Osteoblast and Osteoclast In Vitro Co-Culture Models and Their Translation for Preclinical Drug Testing Applications. Int. J. Mol. Sci..

[29] Buck H.V., Stains J.P. (2024). Osteocyte-Mediated Mechanical Response Controls Osteoblast Differentiation and Function. Front. Physiol..

[30] Oftadeh R., Perez-Viloria M., Villa-Camacho J.C., Vaziri A., Nazarian A. (2015). Biomechanics and Mechanobiology of Trabecular Bone: A Review. J. Biomech. Eng..

[31] Erdal N., Gürgül S., Demirel C., Yildiz A. (2012). The Effect of Insulin Therapy on Biomechanical Deterioration of Bone in Streptozotocin (STZ)-Induced Type 1 Diabetes Mellitus in Rats. Diabetes Res. Clin. Pract..

[32] Shu J., Wang K., Liu Y., Zhang J., Ding X., Sun H., Wu J., Huang B., Qiu J., Sheng H. (2024). Trichosanthin Alleviates Streptozotocin-Induced Type 1 Diabetes Mellitus in Mice by Regulating the Balance between Bone Marrow-Derived IL6+ and IL10+ MDSCs. Heliyon.

[33] Walle M., Duseja A., Whittier D.E., Vilaca T., Paggiosi M., Eastell R., Müller R., Collins C.J. (2024). Bone Remodeling and Responsiveness to Mechanical Stimuli in Individuals with Type 1 Diabetes Mellitus. J. Bone Miner. Res..

[34] Rubin M.R., Dhaliwal R. (2024). Role of Advanced Glycation Endproducts in Bone Fragility in Type 1 Diabetes. Bone.

[35] Tsentidis C., Gourgiotis D., Kossiva L., Marmarinos A., Doulgeraki A., Karavanaki K. (2016). Sclerostin Distribution in Children and Adolescents with Type 1 Diabetes Mellitus and Correlation with Bone Metabolism and Bone Mineral Density: Sclerostin in T1DM Children and Adolescents. Pediatr. Diabetes.

[36] Dhaon P., Shah V. (2014). Type 1 Diabetes and Osteoporosis: A Review of Literature. Indian J. Endocrinol. Metab..

[37] Khan T.S., Fraser L.-A. (2015). Type 1 Diabetes and Osteoporosis: From Molecular Pathways to Bone Phenotype. J. Osteoporos..

[38] Abdulameer S.A., Sulaiman S.A.S., Hassali M.A.A., Subramaniam K., Sahib M.N. (2012). Osteoporosis and Type 2 Diabetes Mellitus: What Do We Know, and What We Can Do?. Patient Prefer. Adherence.

[39] Sheu A., Greenfield J.R., White C.P., Center J.R. (2023). Contributors to Impaired Bone Health in Type 2 Diabetes. Trends Endocrinol. Metab..

[40] Giovos G., Yavropoulou M.P., Yovos J.G. (2019). The Role of Cellular Senescence in Diabetes Mellitus and Osteoporosis: Molecular Pathways and Potential Interventions. Hormones.

[41] Jiang H., Li D., Han Y., Li N., Tao X., Liu J., Zhang Z., Yu Y., Wang L., Yu S. (2023). The Role of Sclerostin in Lipid and Glucose Metabolism Disorders. Biochem. Pharmacol..

[42] Karim L., Rezaee T., Vaidya R. (2019). The Effect of Type 2 Diabetes on Bone Biomechanics. Curr. Osteoporos. Rep..

[43] Eller-Vainicher C., Cairoli E., Grassi G., Grassi F., Catalano A., Merlotti D., Falchetti A., Gaudio A., Chiodini I., Gennari L. (2020). Pathophysiology and Management of Type 2 Diabetes Mellitus Bone Fragility. J. Diabetes Res..

[44] Bonnet N., Bourgoin L., Biver E., Douni E., Ferrari S. (2019). RANKL Inhibition Improves Muscle Strength and Insulin Sensitivity and Restores Bone Mass. J. Clin. Investig..

[45] Sheu A., Greenfield J.R., White C.P., Center J.R. (2022). Assessment and Treatment of Osteoporosis and Fractures in Type 2 Diabetes. Trends Endocrinol. Metab..

[46] Shao X., Tian Y., Liu J., Yan Z., Ding Y., Hao X., Wang D., Shen L., Luo E., Guo X.E. (2024). Rescuing SERCA2 Pump Deficiency Improves Bone Mechano-Responsiveness in Type 2 Diabetes by Shaping Osteocyte Calcium Dynamics. Nat. Commun..

[47] Wong S.K., Mohamad N.V., Jayusman P.A., Ibrahim N. (2023). ‘Izzah A Review on the Crosstalk between Insulin and Wnt/β-Catenin Signalling for Bone Health. Int. J. Mol. Sci..

[48] Cipriani C., Colangelo L., Santori R., Renella M., Mastrantonio M., Minisola S., Pepe J. (2020). The Interplay Between Bone and Glucose Metabolism. Front. Endocrinol..

[49] Fuglsang-Nielsen R., Rakvaag E., Vestergaard P., Hartmann B., Holst J.J., Hermansen K., Gregersen S., Starup-Linde J. (2020). Consumption of Nutrients and Insulin Resistance Suppress Markers of Bone Turnover in Subjects with Abdominal Obesity. Bone.

[50] Yuan S., Wan Z.-H., Cheng S.-L., Michaëlsson K., Larsson S.C. (2021). Insulin-like Growth Factor-1, Bone Mineral Density, and Fracture: A Mendelian Randomization Study. J. Clin. Endocrinol. Metab..

[51] Carpio-Rivera E., Chacón-Araya Y., Moncada-Jiménez J. (2024). Effect of Exercise-Induced Body Fluid Redistribution on Body Composition in Males Using Dual Energy X-Ray Absorptiometry. J. Sports Sci..

[52] Żuchowski P., Jeka D. (2024). Dual-Energy X-Ray Absorptiometry—Is It the Gold Standard, or Is Bone Mineral Density Everything?. Rheumatology.

[53] Ghalenavi E., Mirfeizi Z., Hashemzadeh K., Sahebari M., Joker M.H., Samadi S. (2024). Diagnostic Value of Radiographic Singh Index Compared to Dual-Energy X-Ray Absorptiometry Scan in Diagnosing Osteoporosis: A Systematic Review. Arch. Bone Jt. Surg..

[54] Giuliodori A., Soudah E., Malouf J., Martel-Duguech L., Amodru V., Gil J., Hernández J.A., Domingo M.P., Webb S.M., Valassi E. (2024). Evaluation of Bone-Related Mechanical Properties in Female Patients with Long-Term Remission of Cushing’s Syndrome Using Quantitative Computed Tomography–Based Finite Element Analysis. Eur. J. Endocrinol..

[55] Rasmussen N.H., Dal J., Kvist A.V., Van Den Bergh J.P., Jensen M.H., Vestergaard P. (2023). Bone Parameters in T1D and T2D Assessed by DXA and HR-pQCT—A Cross-Sectional Study: The DIAFALL Study. Bone.

[56] Das L., Laway B.A., Sahoo J., Dhiman V., Singh P., Rao S.D., Korbonits M., Bhadada S.K., Dutta P. (2024). Bone Mineral Density, Turnover, and Microarchitecture Assessed by Second-Generation High-Resolution Peripheral Quantitative Computed Tomography in Patients with Sheehan’s Syndrome. Osteoporos. Int..

[57] Riaz S., Shakil Ur Rehman S., Hafeez S., Hassan D. (2024). Effects of Kinect-Based Virtual Reality Training on Bone Mineral Density and Fracture Risk in Postmenopausal Women with Osteopenia: A Randomized Controlled Trial. Sci. Rep..

[58] Schini M., Johansson H., Harvey N.C., Lorentzon M., Kanis J.A., McCloskey E.V. (2023). An Overview of the Use of the Fracture Risk Assessment Tool (FRAX) in Osteoporosis. J. Endocrinol. Invest..

[59] Stephanus A.D., Ramos S.C.L., Netto O.S., De Carvalho L.S.F., Campos-Staffico A.M. (2024). Fracture Risk Assessment Tool-Based Screening for Osteoporosis in Older Adults in Resource-Limited Settings. J. Clin. Densitom..

[60] Hong J.-M., Kim U.-G., Yeo I.-S.L. (2022). Comparison of Three-Dimensional Digital Analyses and Two-Dimensional Histomorphometric Analyses of the Bone-Implant Interface. PLoS ONE.

[61] Kasahara M., Someya T., Kitamura K., Watanabe G., Matsunaga S., Abe S., Hattori M. (2024). Analysis of Bone Mineral Density and Bone Quality of Cortical Bone in the Human Hyoid Body and Histological Observation of the Entheses. J. Funct. Biomater..

[62] Loundagin L.L., Harrison K.D., Wei X., Cooper D.M.L. (2024). Understanding Basic Multicellular Unit Activity in Cortical Bone through 3D Morphological Analysis: New Methods to Define Zones of the Remodeling Space. Bone.

[63] Qiu S., Dhaliwal R., Divine G., Warner E., Rao S.D. (2024). Differences in Bone Histomorphometry between White Postmenopausal Women with and without Atypical Femoral Fracture after Long-Term Bisphosphonate Therapy. J. Bone Miner. Res..

[64] Wojciechowska-Puchałka J., Calik J., Krawczyk J., Obrzut J., Tomaszewska E., Muszyński S., Wojtysiak D. (2024). The Effect of Caponization on Tibia Bone Histomorphometric Properties of Crossbred Roosters. Sci. Rep..

[65] Coutts L.V., Jenkins T., Li T., Dunlop D.G., Oreffo R.O.C., Cooper C., Harvey N.C., Thurner P.J., Arden N.K., Latham J.M. (2015). Variability in Reference Point Microindentation and Recommendations for Testing Cortical Bone: Location, Thickness and Orientation Heterogeneity. J. Mech. Behav. Biomed. Mater..

[66] Arnold M., Zhao S., Ma S., Giuliani F., Hansen U., Cobb J.P., Abel R.L., Boughton O. (2017). Microindentation—A Tool for Measuring Cortical Bone Stiffness?: A Systematic Review. Bone Jt. Res..

[67] Rudäng R., Zoulakis M., Sundh D., Brisby H., Diez-Perez A., Johansson L., Mellström D., Darelid A., Lorentzon M. (2016). Bone Material Strength Is Associated with Areal BMD but Not with Prevalent Fractures in Older Women. Osteoporos. Int..

[68] Malgo F., Hamdy N.A.T., Papapoulos S.E., Appelman-Dijkstra N.M. (2015). Bone Material Strength as Measured by Microindentation In Vivo Is Decreased in Patients With Fragility Fractures Independently of Bone Mineral Density. J. Clin. Endocrinol. Metab..

[69] Al Jofi F.E., Ma T., Guo D., Schneider M.P., Shu Y., Xu H.H.K., Schneider A. (2018). Functional Organic Cation Transporters Mediate Osteogenic Response to Metformin in Human Umbilical Cord Mesenchymal Stromal Cells. Cytotherapy.

[70] Tanaka M., Inoue H., Takahashi N., Uehara M. (2023). AMPK Negatively Regulates RANKL-Induced Osteoclast Differentiation by Controlling Oxidative Stress. Free Radic. Biol. Med..

[71] Xin Y., Zhao N., Wang Y. (2022). Multiple Roles of Runt-Related Transcription Factor-2 in Tooth Eruption: Bone Formation and Resorption. Arch. Oral Biol..

[72] Yahiro Y., Maeda S., Morikawa M., Koinuma D., Jokoji G., Ijuin T., Komiya S., Kageyama R., Miyazono K., Taniguchi N. (2020). BMP-Induced Atoh8 Attenuates Osteoclastogenesis by Suppressing Runx2 Transcriptional Activity and Reducing the Rankl/Opg Expression Ratio in Osteoblasts. Bone Res..

[73] Arafa E.-S.A., Elgendy N.O., Elhemely M.A., Abdelaleem E.A., Mohamed W.R. (2023). Diosmin Mitigates Dexamethasone-Induced Osteoporosis in Vivo: Role of Runx2, RANKL/OPG, and Oxidative Stress. Biomed. Pharmacother..

[74] Jiating L., Buyun J., Yinchang Z. (2019). Role of Metformin on Osteoblast Differentiation in Type 2 Diabetes. BioMed Res. Int..

[75] Blümel J.E., Arteaga E., Aedo S., Arriola-Montenegro J., López M., Martino M., Miranda C., Miranda O., Mostajo D., Ñañez M. (2020). Metformin Use Is Associated with a Lower Risk of Osteoporosis in Adult Women Independent of Type 2 Diabetes Mellitus and Obesity. REDLINC IX Study. Gynecol. Endocrinol..

[76] Shaik A.R., Singh P., Shaik C., Kohli S., Vohora D., Ferrari S.L. (2021). Metformin: Is It the Well Wisher of Bone Beyond Glycemic Control in Diabetes Mellitus?. Calcif. Tissue Int..

[77] De Vries T.J., Kleemann A.S., Jin J., Schoenmaker T. (2023). The Differential Effect of Metformin on Osteocytes, Osteoblasts, and Osteoclasts. Curr. Osteoporos. Rep..

[78] Ma L., Wu X., Ling-Ling E., Wang D.-S., Liu H.-C. (2009). The Transmembrane Transport of Metformin by Osteoblasts from Rat Mandible. Arch. Oral Biol..

[79] Salari-Moghaddam A., Sadeghi O., Keshteli A.H., Larijani B., Esmaillzadeh A. (2019). Metformin Use and Risk of Fracture: A Systematic Review and Meta-Analysis of Observational Studies. Osteoporos. Int..

[80] Zhao J., Li Y., Zhang H., Shi D., Li Q., Meng Y., Zuo L. (2019). Preventative Effects of Metformin on Glucocorticoid-Induced Osteoporosis in Rats. J. Bone Miner. Metab..

[81] Yang B.R., Cha S.H., Lee K.E., Kim J.W., Lee J., Shin K.-H. (2021). Effect of Dipeptidyl Peptidase IV Inhibitors, Thiazolidinedione, and Sulfonylurea on Osteoporosis in Patients with Type 2 Diabetes: Population-Based Cohort Study. Osteoporos. Int..

[82] Zeng Z., Huang S.-Y., Sun T. (2020). Pharmacogenomic Studies of Current Antidiabetic Agents and Potential New Drug Targets for Precision Medicine of Diabetes. Diabetes Ther..

[83] Leiter L.A. (2020). Latest Evidence on Sulfonylureas: What’s New?. Diabetes Ther..

[84] Kumari C., Yagoub G., Ashfaque M., Jawed S., Hamid P. (2021). Consequences of Diabetes Mellitus in Bone Health: Traditional Review. Cureus.

[85] Al-Saleh Y., Sabico S., Al-Furqani A., Jayyousi A., Alromaihi D., Ba-Essa E., Alawadi F., Alkaabi J., Hassanein M., Al-Sifri S. (2021). Sulfonylureas in the Current Practice of Type 2 Diabetes Management: Are They All the Same? Consensus from the Gulf Cooperation Council (GCC) Countries Advisory Board on Sulfonylureas. Diabetes Ther..

[86] Yen F.-S., Wei J.C.-C., Yu T.-S., Hsu C.Y., Hsu C.-C., Hwu C.-M. (2022). Sulfonylurea Use in Patients with Type 2 Diabetes and COPD: A Nationwide Population-Based Cohort Study. Int. J. Environ. Res. Public Health.

[87] Proks P., Reimann F., Green N., Gribble F., Ashcroft F. (2002). Sulfonylurea Stimulation of Insulin Secretion. Diabetes.

[88] Ha J., Lim Y., Kim M.K., Kwon H.-S., Song K.-H., Ko S.H., Kang M.I., Moon S.D., Baek K.-H. (2021). Comparison of the Effects of Various Antidiabetic Medication on Bone Mineral Density in Patients with Type 2 Diabetes Mellitus. Endocrinol. Metab..

[89] Zhang Z., Cao Y., Tao Y., E M., Tang J., Liu Y., Li F. (2020). Sulfonylurea and Fracture Risk in Patients with Type 2 Diabetes Mellitus: A Meta-analysis. Diabetes Res. Clin. Pract..

[90] Tao Y., E M., Shi J., Zhang Z. (2021). Sulfonylureas Use and Fractures Risk in Elderly Patients with Type 2 Diabetes Mellitus: A Meta-Analysis Study. Aging Clin. Exp. Res..

[91] Hannele Y.-J. (2004). Thiazolidinediones. N. Engl. J. Med..

[92] Hurren K.M., Dunham M.W. (2021). Are Thiazolidinediones a Preferred Drug Treatment for Type 2 Diabetes?. Expert Opin. Pharmacother..

[93] Long N., Le Gresley A., Wren S.P. (2021). Thiazolidinediones: An In–Depth Study of Their Synthesis and Application to Medicinal Chemistry in the Treatment of Diabetes Mellitus. ChemMedChem.

[94] Köseoğlu D., Take G., Yılmaz B.A., Kan E., Çakır N. (2021). The Effect of Diabetes Mellitus, Insulin, and Thiazolidinediones on Bone Histomorphometry in Streptozotocin-Induced Diabetic Postmenopausal Wistar Rats. Sudan J. Med. Sci..

[95] Wang L.H., Yang X.Y., Zhang X., Huang J., Hou J., Li J., Xiong H., Mihalic K., Zhu H., Xiao W. (2004). Transcriptional Inactivation of STAT3 by PPARγ Suppresses IL-6-Responsive Multiple Myeloma Cells. Immunity.

[96] Hou X., Tian F. (2022). STAT3-Mediated Osteogenesis and Osteoclastogenesis in Osteoporosis. Cell Commun. Signal..

[97] Sauer S. (2015). Ligands for the Nuclear Peroxisome Proliferator-Activated Receptor Gamma. Trends Pharmacol. Sci..

[98] Zhang Y., Zhan R.-X., Chen J.-Q., Gao Y., Chen L., Kong Y., Zhong X.-J., Liu M.-Q., Chu J.-J., Yan G.-Q. (2015). Pharmacological Activation of PPAR Gamma Ameliorates Vascular Endothelial Insulin Resistance via a Non-Canonical PPAR Gamma-Dependent Nuclear Factor-Kappa B Trans-Repression Pathway. Eur. J. Pharmacol..

[99] Giglio R.V., Papanas N., Rizvi A.A., Ciaccio M., Patti A.M., Ilias I., Pantea Stoian A., Sahebkar A., Janez A., Rizzo M. (2022). An Update on the Current and Emerging Use of Thiazolidinediones for Type 2 Diabetes. Medicina.

[100] Benova A., Ferencakova M., Bardova K., Funda J., Prochazka J., Spoutil F., Cajka T., Dzubanova M., Balcaen T., Kerckhofs G. (2022). Novel Thiazolidinedione Analog Reduces a Negative Impact on Bone and Mesenchymal Stem Cell Properties in Obese Mice Compared to Classical Thiazolidinediones. Mol. Metab..

[101] Chen R.-D., Yang C.-W., Zhu Q.-R., Li Y., Hu H.-F., Wang D.-C., Han S.-J. (2023). Comparison of the Effects of Metformin and Thiazolidinediones on Bone Metabolism: A Systematic Review and Meta-Analysis. Medicina.

[102] Wei W., Wan Y. (2011). Thiazolidinediones on PPAR *γ*: The Roles in Bone Remodeling. PPAR Res..

[103] Yau H., Rivera K., Lomonaco R., Cusi K. (2013). The Future of Thiazolidinedione Therapy in the Management of Type 2 Diabetes Mellitus. Curr. Diab. Rep..

[104] Boer G.A., Holst J.J. (2020). Incretin Hormones and Type 2 Diabetes—Mechanistic Insights and Therapeutic Approaches. Biology.

[105] Kitaura H., Ogawa S., Ohori F., Noguchi T., Marahleh A., Nara Y., Pramusita A., Kinjo R., Ma J., Kanou K. (2021). Effects of Incretin-Related Diabetes Drugs on Bone Formation and Bone Resorption. Int. J. Mol. Sci..

[106] Daniilopoulou I., Vlachou E., Lambrou G.I., Ntikoudi A., Dokoutsidou E., Fasoi G., Govina O., Kavga A., Tsartsalis A.N. (2022). The Impact of GLP1 Agonists on Bone Metabolism: A Systematic Review. Medicina.

[107] Dicembrini I., Mannucci E., Rotella C.M. (2012). Bone: Incretin Hormones Perceiver or Receiver?. Exp. Diabetes Res..

[108] Ali A., Flatt P.R., Irwin N. (2024). Gut-Derived Peptide Hormone Analogues and Potential Treatment of Bone Disorders in Obesity and Diabetes Mellitus. Clin. Med. Insights Endocrinol. Diabetes.

[109] Mabilleau G., Gobron B., Bouvard B., Chappard D. (2018). Incretin-Based Therapy for the Treatment of Bone Fragility in Diabetes Mellitus. Peptides.

[110] Makrilakis K. (2019). The Role of DPP-4 Inhibitors in the Treatment Algorithm of Type 2 Diabetes Mellitus: When to Select, What to Expect. Int. J. Environ. Res. Public Health.

[111] Kawanami D., Takashi Y., Takahashi H., Motonaga R., Tanabe M. (2021). Renoprotective Effects of DPP-4 Inhibitors. Antioxidants.

[112] Kanasaki K. (2018). The Role of Renal Dipeptidyl Peptidase-4 in Kidney Disease: Renal Effects of Dipeptidyl Peptidase-4 Inhibitors with a Focus on Linagliptin. Clin. Sci..

[113] Deacon C.F. (2019). Physiology and Pharmacology of DPP-4 in Glucose Homeostasis and the Treatment of Type 2 Diabetes. Front. Endocrinol..

[114] Chitadze G., Wehkamp U., Janssen O., Brüggemann M., Lettau M. (2021). The Serine Protease CD26/DPP4 in Non-Transformed and Malignant T Cells. Cancers.

[115] Saini K., Sharma S., Khan Y. (2023). DPP-4 Inhibitors for Treating T2DM—Hype or Hope? An Analysis Based on the Current Literature. Front. Mol. Biosci..

[116] Monami M., Dicembrini I., Antenore A., Mannucci E. (2011). Dipeptidyl Peptidase-4 Inhibitors and Bone Fractures. Diabetes Care.

[117] Yang Y., Zhao C., Liang J., Yu M., Qu X. (2017). Effect of Dipeptidyl Peptidase-4 Inhibitors on Bone Metabolism and the Possible Underlying Mechanisms. Front. Pharmacol..

[118] Driessen J.H.M., Van Onzenoort H.A.W., Henry R.M.A., Lalmohamed A., Van Den Bergh J.P., Neef C., Leufkens H.G.M., De Vries F. (2014). Use of Dipeptidyl Peptidase-4 Inhibitors for Type 2 Diabetes Mellitus and Risk of Fracture. Bone.

[119] Nair S., Wilding J.P.H. (2010). Sodium Glucose Cotransporter 2 Inhibitors as a New Treatment for Diabetes Mellitus. J. Clin. Endocrinol. Metab..

[120] Hsia D.S., Grove O., Cefalu W.T. (2017). An Update on Sodium-Glucose Co-Transporter-2 Inhibitors for the Treatment of Diabetes Mellitus. Curr. Opin. Endocrinol. Diabetes Obes..

[121] Erythropoulou-Kaltsidou A., Polychronopoulos G., Tziomalos K. (2020). Sodium-Glucose Co-Transporter 2 Inhibitors and Fracture Risk. Diabetes Ther..

[122] Chai S., Liu F., Yang Z., Yu S., Liu Z., Yang Q., Sun F. (2022). Risk of Fracture With Dipeptidyl Peptidase-4 Inhibitors, Glucagon-like Peptide-1 Receptor Agonists, or Sodium-Glucose Cotransporter-2 Inhibitors in Patients With Type 2 Diabetes Mellitus: A Systematic Review and Network Meta-Analysis Combining 177 Randomized Controlled Trials With a Median Follow-Up of 26 Weeks. Front. Pharmacol..

[123] Zhuo M., Hawley C.E., Paik J.M., Bessette L.G., Wexler D.J., Kim D.H., Tong A.Y., Kim S.C., Patorno E. (2021). Association of Sodium-Glucose Cotransporter–2 Inhibitors With Fracture Risk in Older Adults With Type 2 Diabetes. JAMA Netw. Open.

[124] Seguna D., Fava S. (2024). Emerging Trends in Diabetes: An Update on the Role of Sodium-Glucose Co-Transporter 2 Inhibitors. Malta Med. J..

[125] Ye Y., Zhao C., Liang J., Yang Y., Yu M., Qu X. (2019). Effect of Sodium-Glucose Co-Transporter 2 Inhibitors on Bone Metabolism and Fracture Risk. Front. Pharmacol..

[126] Wikarek A., Grabarczyk M., Klimek K., Janoska-Gawrońska A., Suchodolska M., Holecki M. (2024). Effect of Drugs Used in Pharmacotherapy of Type 2 Diabetes on Bone Density and Risk of Bone Fractures. Medicina.

[127] Zhang X., Xing H., Qi F., Liu H., Gao L., Wang X. (2020). Local Delivery of Insulin/IGF-1 for Bone Regeneration: Carriers, Strategies, and Effects. Nanotheranostics.

[128] Clowes J.A., Robinson R.T., Heller S.R., Eastell R., Blumsohn A. (2002). Acute Changes of Bone Turnover and PTH Induced by Insulin and Glucose: Euglycemic and Hypoglycemic Hyperinsulinemic Clamp Studies. J. Clin. Endocrinol. Metab..

[129] Liu H., Liu L., Rosen C.J. (2024). PTH and the Regulation of Mesenchymal Cells within the Bone Marrow Niche. Cells.

[130] Fan Y., Hanai J., Le P.T., Bi R., Maridas D., DeMambro V., Figueroa C.A., Kir S., Zhou X., Mannstadt M. (2017). Parathyroid Hormone Directs Bone Marrow Mesenchymal Cell Fate. Cell Metab..

[131] Fulzele K., Clemens T.L. (2012). Novel Functions for Insulin in Bone. Bone.

[132] Sheu A., White C.P., Center J.R. (2024). Bone Metabolism in Diabetes: A Clinician’s Guide to Understanding the Bone–Glucose Interplay. Diabetologia.

